# Ulnar paddlefish carpometacarpal dislocation of the three lesser fingers: a case report

**DOI:** 10.11604/pamj.2017.28.155.12517

**Published:** 2017-10-18

**Authors:** Lassaad Hassini, Thabet Mouelhi, Mohamed Ali Khalifa, Mourad Mtaoumi, Mohamed Laaziz Ben Ayeche

**Affiliations:** 1 Department of Orthopaedic Surgery, University Hospital Sahloul, Sousse, Tunisia

**Keywords:** Carpometacarpal dislocation, paddlefish, ulnar, reduction

## Abstract

The long fingers’ paddlefish carpometacarpal (CMC) dislocation is exceptional. Most dislocations occur after high energy trauma. Untreated, these lesions can result in chronic instability of the CMC joints and early osteoarthritis. We report the case of a 20-year-old patient presenting with an ulnar paddlefish CMC fracture-dislocation of the three lesser fingers resulting from a hand trauma in the context of an occupational accident. Treatment is usually surgical though no strict consensus can be found upon literature review. If diagnosed early and no associated fractures are found, CMC dislocation could benefit from conservative treatment.

## Introduction

Multiple dislocations of the carpometacarpal (CMD) are rare. The one involving the 2^nd^ or 3^rd^ metacarpal is poorly described in literature [1]. They occur most often after a high-energy trauma in young adults. The main etiology remains traffic accidents where massive fractures are often associated with the dislocation because of the high velocity of the trauma. These injuries seriously compromise the functional prognosis. Delayed treatment can result in neurovascular injuries due to oedema and prolonged compression. Untreated, these lesions can result in chronic instability of the CMC joints and early articular degeneration. We report the case of a 20-year-old female patient with an ulnar paddlefish CMD of the three lesser fingers.

## Patient and observation

**Figure 1:**
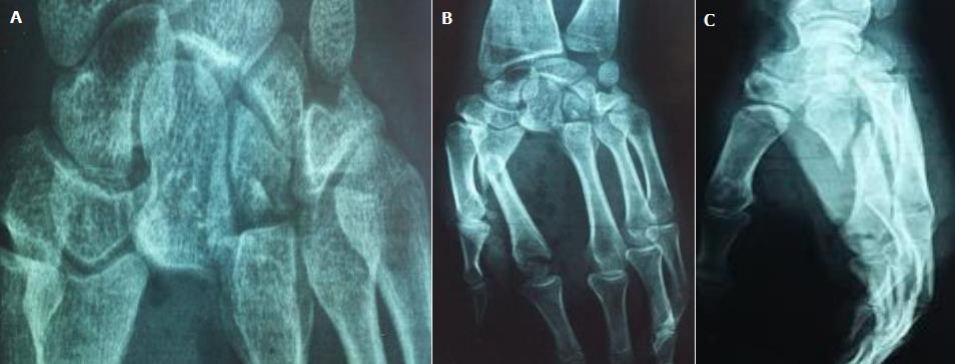
(A, B, C) X-ray of the left hand (front + profile) showed a dislocation of the three lesser fingers associated with fracture of the hamatum and the second metacarpal. The three lesser metacarpals and the distal fragment of the hamatum were medially displaced

**Figure 2:**
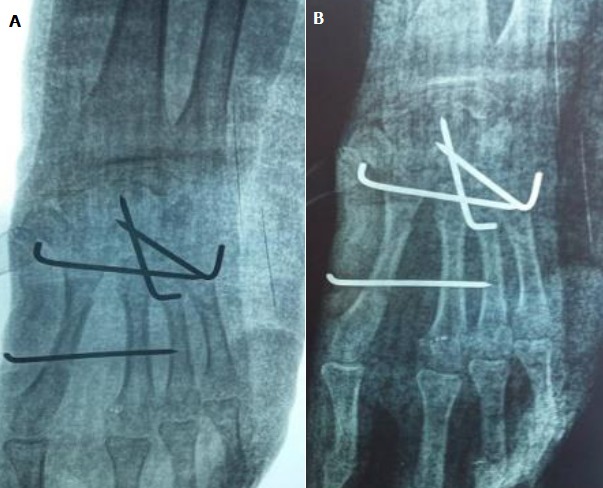
(A, B) post-operative X ray of the left hand

**Figure 3:**
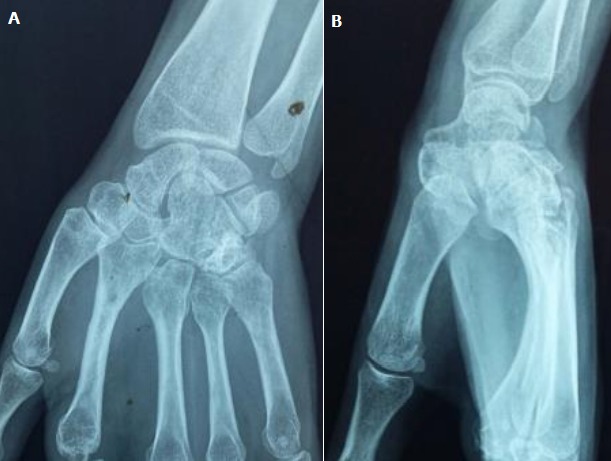
(A, B) follow-up X ray of the left hand (front + profile) one year later

A 20-year-old right-handed female factory worker with nomedical history is consulting in the emergency ward with an open trauma of the left hand resulting from an occupational accident. Her hand was crushed under a heavy object. Physical examination found an important swelling and obvious distortion at the dorsum of the hand associated with wounds of the ulnar and radial edges of the left hand. The neurovascular examination was normal; in particular no sensitive deficit in the median nerve area was noted. Plain radiographs with front and lateral views showed a dislocation of the three lesser fingers associated with fractures of the hamatum and the second metacarpal (Figure 1). The three lesser metacarpals and the distal fragment of the hamatum were medially displaced. The patient was immediately brought to the operating theatre. The treatment consisted of a reduction and stabilisation using a multiple carpo-metacarpal and cross inter-metacarpal pinning (Figure 2). The wound was cleaned up trimmed and sutured. The had and the wrist were immobilized with a splint for six weeks. The patient had an intense and regular rehabilitation program. At one year follow-up, the outcome is good (Figure 3): the patient is painless with good bone consolidation in right position, strictly normal range of motion (metacarpophalangeal 90°, proximal interphalagienne 100° and distal interphalageal 90°) and a 80% grip strength compared to the right side.

## Discussion

Longitudinal volar CMC ligaments finger joints are vulnerable because of their anatomical location. Lesions of long fingers carpometacarpal joints are rare and represent less than 0.31% of all hand injuries [1-5]. About 300 cases are reported in the literature. Isolated dislocation of the 4^th^ or 5^th^ metacarpal is the best known and explained by the traditional opposition between the relative mobility of the metacarpal block 4-5 and the fixity of Block 2-3 [2, 5]. The ulnar paddlefish CMD of the three lesser long fingers is exceptional. It usually affects young adults. Traffic accidents and high velocity injuries are the main etiologies. The most widely used lesional classification is the one described by Costagliola et al. As for the foot, it individualises two architectural entities: the column represented by the thumb and the paddlefish represented by the four lesser fingers. Complete paddlefish dislocations (35% of cases) are more frequently dorsal (85%) than palmar or divergent [4]. They are usually associated with fractures of the proximal phalanges, metacarpals or carpal indicating the violence of the trauma. The diagnosis is not easy, which may result in delayed management [6] (Guimaraes: 5/26, Gangloff 9/31) [7]. The mechanism can be evocative, but the clinical examination typically found a “large painful hand” with functional impairment, without specific sign. The diagnosis is always radiological. Usually diagnosed with a true lateral view X-ray of the hand. In our case report anteroposterior X-rays led to the diagnostics.

The plain radiographs search indirect signs of CMD: loss of CM joint space decreased medial carp height and disharmony of the Gilula arc III on the anterior-posterior view, increased carp palmodorsal diameter on strict lateral view [6]. Additionally, oblique radiographs of the hand can be useful to demonstrate CMC dislocation [8]. Moreover, Allieu recommends a CT scan for better analysis of the dislocation [9] and the diagnosis of possible carpal or associated osteochondral lesion, undetected during the interpretation of plain radiographs. These injuries should be treated at once because of the risk of intractability, cutaneous complications, as well as vascular and nerve compression in the most severe cases. No consensus was released from the literature regarding treatment. According to Benoit et al, the simple external reduction should be abandoned for the incidence of secondary displacement after regression of the oedema [10]. Some secondary dislocations after treatment by closed reduction and splint immobilisation have been described, occurring within two weeks of the reduction [8]. All authors emphasize on the surgical fixation of the carpal-metacarpal dislocation with pinning because of its reliability to maintain the reduction [2, 3, 5, 8]. Therefore, X-rays of the hand are recommended during follow-up. Rehabilitation is a critical time in dealing with these complex lesions. Two studies have investigated the long term outcome (respectively 6.5 years and 41 months later) after surgical treatment of the CMD. The conclusion of these studies was the lack of correlation between the postoperative outcome and the type of dislocation, the mechanism of injury, the reduction by external or direct internal manipulation, the pin holding time and the immobilization duration [6].

## Conclusion

The CMD of the long fingers are extremely rare lesions whose diagnosis is mainly based on radiographs in a suggestive context. Many go unnoticed. CMC dislocations can easy be underdiagnosed if clinical signs are overlooked. A quick and appropriate treatment allows a painless recovery of functional mobility with a clamping force near normal. Unrecognized CMC dislocation can lead to neurovascular injuries as well as chronic instability and early articular degeneration. However, a long-term evaluation is needed to assess the stability of this result over time.

## Competing interests

The authors declare no competing interests.
